# Sulodexide pretreatment attenuates renal ischemia-reperfusion injury in rats

**DOI:** 10.18632/oncotarget.14309

**Published:** 2016-12-27

**Authors:** Jianyong Yin, Weibin Chen, Fenfen Ma, Zeyuan Lu, Rui Wu, Guangyuan Zhang, Niansong Wang, Feng Wang

**Affiliations:** ^1^ Department of Nephrology, Shanghai Eighth People's Hospital, Shanghai Jiao Tong University Affiliated Sixth People's Hospital, Shanghai 200233, China; ^2^ Department of Laboratory Medicine, Shanghai Jiao Tong University Affiliated Sixth People's Hospital, Shanghai 200233, China; ^3^ Department of Pharmacy, Shanghai Pudong Hospital, Shanghai 201399, China; ^4^ Department of Urology, Affiliated Zhongda Hospital of Southeast University, Nanjing 210009, China

**Keywords:** sulodexide, ischemia-reperfusion, antithrombin III, oxidative stress, apoptosis

## Abstract

Sulodexide is a potent antithrombin agent, however, whether it has beneficial effects on renal ischemia-reperfusion injury (IRI) remains unknown. In the present study, we assessed the therapeutic effects of sulodexide in renal IRI and tried to investigate the potential mechanism. One dose of sulodexide was injected intravenously in Sprague-Dawley rats 30 min before bilateral kidney ischemia for 45 min. The animals were sacrificed at 3h and 24h respectively. Our results showed that sulodexide pretreatment improved renal dysfunction and alleviated tubular pathological injury at 24h after reperfusion, which was accompanied with inhibition of oxidative stress, inflammation and cell apoptosis. Moreover, we noticed that antithrombin III (ATIII) was activated at 3h after reperfusion, which preceded the alleviation of renal injury. For *in vitro* study, hypoxia/reoxygenation (H/R) injury model for HK2 cells was carried out and apoptosis and reactive oxygen species (ROS) levels were evaluated after sulodexide pretreatment. Consistently, sulodexide pretreatment could reduce apoptosis and ROS level in HK2 cells under H/R injury. Taken together, sulodexide pretreatment might attenuate renal IRI through inhibition of inflammation, oxidative stress and apoptosis, and activation of ATIII.

## INTRODUCTION

Renal ischemia reperfusion injury (IRI) is commonly seen in various clinical settings such as kidney transplantation, hemorrhagic shock or cardiovascular surgery [1]. Although numerous efforts had been made to avoid or alleviate renal IRI, the morbidity and mortality of ischemic acute kidney injury (AKI) still remains high [2]. Therefore, it is urgent to identify novel preventive strategies to decrease AKI incidence and to improve clinical outcome.

Currently, the exact pathophysiological mechanism of AKI still remains elusive. However, it has been established that the pathophysiology of AKI predominantly involves continued hypoperfusion, inflammation, oxidative stress, and tubular epithelial apoptosis [3–5]. Recently, several studies have shown that microvascular thrombosis generation also plays a pivotal role in the pathophysiology of AKI, and many antithrombin agents, such as heparin [6] and antithrombin III (ATIII) [7–9] could mitigate renal IRI. Therefore, pharmacological agents with multiple function such as anti-coagulation, anti-oxidative and anti-inflammation properties may be promising preventative strategies for AKI.

Among various candidates, sulodexide, a purified mixture of glycosaminoglycan composed of low molecular weight heparin and dermatan sulfate [10], has been reported to exert its reno-protective effect in many renal diseases [11–14]. In addition to its anti-coagulant function, it was reported that sulodexide has anti-oxidative effects [15, 16] and anti-inflammatory [17, 18], as well as the anti-ischemic effects [19]. Previous studies have demonstrated that sulodexide attenuated diabetic nephropathy through inhibiting cell proliferation and decreasing matrix accumulation [20]. Given the effects of sulodexide, it is reasonable to speculate that sulodexide administration may be able to mitigate renal IRI. In the present study, we examined the therapeutic effect of sulodexide administration on renal IRI in rats and tubular epithelial cells. Furthermore, the potential mechanisms were investigated.

## RESULTS

### Sulodexide administration mitigated renal ischemia-reperfusion injury

**Figure 1:**
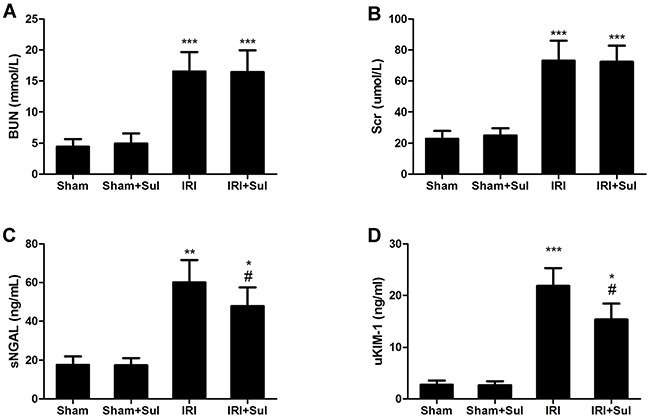
Sulodexide reduced renal ischemia-reperfusion injury in rats Rats were challenged with sham-operation or 45 minutes of bilateral renal ischemia, respectively. Blood and urine were harvested at 24 h after reperfusion. **A**. Blood urea nitrogen. **B**. Serum creatinine. **C**. Serum NGAL. **D**. Urinary KIM-1. Sham, sham-operated group with vehicle injection; Sham+Sul, sham-operated group with Sulodexide injection; IRI, IRI group with vehicle injection; IRI+Sul, IRI with sulodexide injection. All data were presented as means ± SD (n=6). *P<0.05 versus sham,^*^P<0.01 versus sham, ^**^*P<0.001 versus sham; ^#^*P*<0.05 versus IRI.

As shown in Figure 1A-1B, rats in IRI groups displayed significant exacerbation of renal function 24h after reperfusion, as indicated by remarkably increased levels of Scr and BUN compared with that in sham rats. However, the levels of Scr and BUN were significantly decreased in IRI rats pre-treated with sulodexide (Scr, IRI vs. IRI+Sul, *P*<0.05; BUN, IRI+sulodexide vs. IRI+Sul, *P*<0.05). In agreement with the alteration of Scr and BUN, the concentrations of sNGAL and uKIM-1, as the more precise and sensitive marker for diagnosing AKI, were also lower in sulodexide-administered IRI rats than that in un-treated IRI rats. Collectively, these data suggested that sulodexide was able to protect against ischemia-reperfusion kidney injury.

### Sulodexide attenuated morphological change after ischemia-reperfusion injury

**Figure 2:**
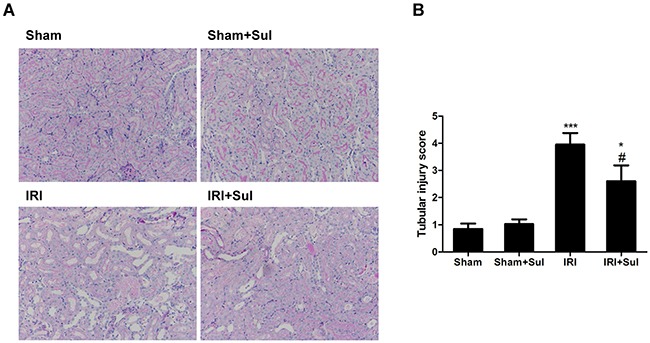
Sulodexide pretreatment mitigated renal histological injury in IRI rats Rats were challenged with sham-operation or 45 minutes of bilateral renal ischemia, respectively. Kidney tissues were harvested at 24 h after reperfusion. Periodic acid–Schiff (PAS) staining and a semi-quantitative scoring system was used to evaluate the severity of tubular injury. **A**. Representative images of renal PAS staining (magnification, 200×). **B**. Semi-quantitative assessment of tubular injury. All data were presented as means ± SD (n=6). *P<0.05 versus Sham, ^**^*P<0.001 versus Sham; ^#^*P*<0.05 versus IRI.

To evaluate the extent of kidney pathological injury, kidney sections were stained with PAS. As expected, ischemia-reperfusion led to typical tubular injury characterized by pronounced renal tubular detachment, luminal congestion with loss of brush border, tubular cell necrosis and intratubular cast formation whereas the aforementioned pathological changes were remarkably alleviated in sulodexide-administered IRI rats, as shown in Figure 2. Meanwhile, the pathological score of histological lesions was significantly lower in sulodexide-treated IRI rats (2.60±0.58) than un-treated IRI rats (3.95±0.43) at 24h after reperfusion.

### Effects of sulodexide on oxidative stress and inflammation in kidney tissues

**Figure 3:**
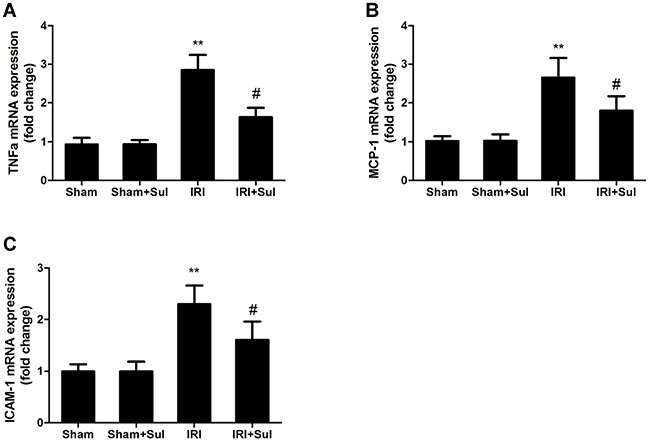
Sulodexide suppressed the expression of pro-inflammatory cytokines in kidney Kidney tissues were harvested at 24 h after reperfusion. **A**. TNFα mRNA expression. **B**. MCP-1 mRNA expression. **C**. ICAM-1 mRNA expression. All data were presented as means ± SD (n=6).^*^P<0.01 versus Sham; ^#^*P*<0.05 versus IRI.

**Figure 4:**
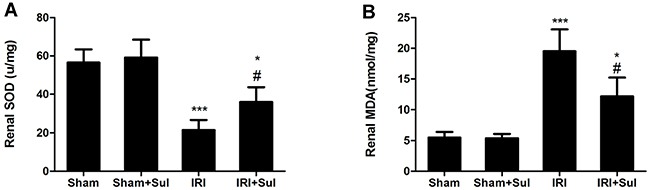
Effects of Sulodexide administration on oxidative stress level in renal tissues Kidney tissues were harvested at 24 h after reperfusion. **A**. Renal MDA levels. **B**. Renal SOD levels. All data were presented as means ± SD (n=6).*P<0.05 versus Sham, ^**^*P<0.001 versus Sham; ^#^*P*<0.05 versus IRI.

To explore whether the reno-protection conferred by sulodexide in IRI was associated with oxidative stress and inflammation, the related markers of oxidative stress and inflammation were examined. As shown in Figure 3A-3B, renal SOD activity was decreased while MDA levels were increased in IRI rats compared with the sham-operated group. Pretreatment with sulodexide restored renal SOD levels and decreased renal MDA levels, suggesting that sulodexide could attenuate oxidative stress from two directions in rats with IRI. Moreover, we found that the renal mRNA expression levels of tumor necrosis factor α (TNFα), monocyte chemotactic protein 1 (MCP-1) and intercellular cell adhesion molecule-1 (ICAM-1) in IRI rats were substantially increased compared with sham rats, which was blunted by sulodexide pretreatment (Figure 4). In summary, sulodexide might exert its reno-protective effects against IRI via inhibiting oxidative stress and inflammatory response.

### Cell apoptosis were alleviated in IRI rats with sulodexide pretreatment

**Figure 5:**
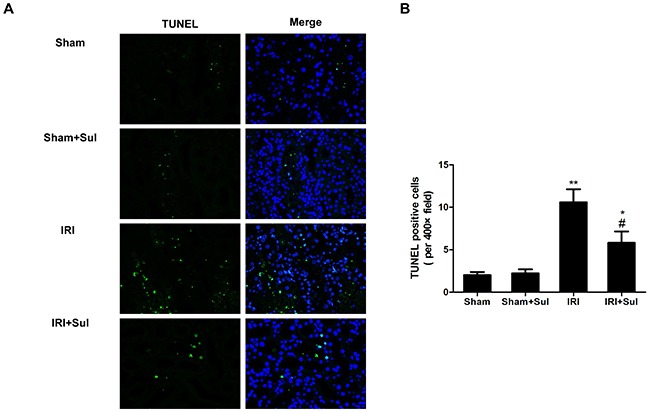
Renal apoptosis was mitigated in sulodexide-administered IRI rats Kidney section harvested at 24 h after reperfusion were stained with terminal transferase-mediated dUTP nick-end labeling (TUNEL). **A**. representative pictures of TUNEL staining (magnification, ×400). **B**. Quantitative analysis of the number of apoptoticcells per field. All data were presented as means ± SD (n=6). *P<0.05 versus Sham, ^*^P<0.01 versus Sham; ^#^*P*<0.05 versus IRI.

**Figure 6:**
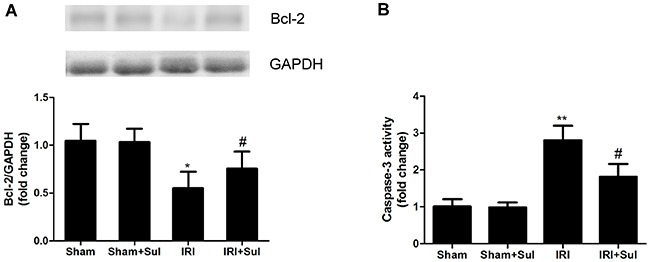
Sulodexide influenced Bcl-2 expression and caspase-3 activity in IRI rats. Kidney tissues were harvested at 24 h after reperfusion **A**. Renal Bcl-2 protein expression. **B**. Changes of caspase-3 activity. Data were presented as means ± SD (n=6).*P<0.05 versus Sham, ^*^P<0.01 versus Sham; ^#^*P*<0.05 versus IRI.

To determine whether sulodexide's beneficial effects on IRI were associated with apoptosis inhibition, we performed TUNEL assays on the kidney sections. In comparison to sham-operated rats, ischemia-reperfusion resulted in elevated apoptosis, and sulodexide could significantly decrease tubular cells apoptosis in IRI rats (Figure 5). Furthermore, the expression of anti-apoptosis protein Bcl-2 and the activity of caspase-3 was also examined. Similarly, it was observed that ischemia-reperfusion led to a substantial decrease in the expression of Bcl-2 and increase in caspase-3 activity as indicated in Figure 6. Our data proved that Bcl-2 expression was restored nearly to normal levels by sulodexide preconditioning while caspase-3 activity was dramatically suppressed. Taken together, sulodexide administration mitigated renal cell apoptosis in IRI rats.

### Antithrombin III was activated before alleviation for renal IRI triggered by sulodexide

**Figure 7:**
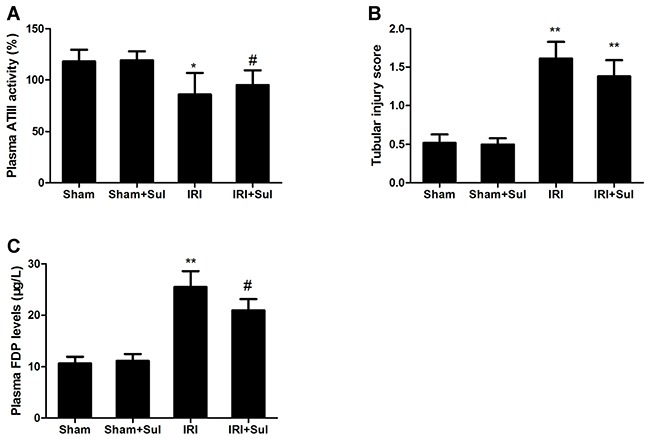
Effects of sulodexide on antithrombin III activity and renal function at the early phase of acute kidney injury Blood were obtained at 3h and 24h after reperfusion. **A**. Plasma ATIII activity at 3h. **B**. Tubular injury at 3h. **C**. Serum FDPs level at 24h. Data were presented as means ± SD (n=6).*P<0.05 versus Sham, ^*^P<0.01 versus Sham; ^#^*P*<0.05 versus IRI.

To determine whether the beneficial effect of sulodexide was dependent on its anti-coagulation function, we detected plasma ATIII activity at 3h after renal reperfusion. Our data revealed that the plasma ATIII activity was declined in IRI animals compared with sham, but was restored approximately to normal levels in sulodexide-treated IRI animals at 3h after reperfusion as shown in Figure 7A. In contrast, there was no significant improvement of renal pathological injury score between sulodexide-treated IRI group (1.38±0.21) and vehicle-treated IRI group (1.61±0.21) at 3h after renal reperfusion (Figure 7B). In addition, we also examined plasma levels of fibrinogen degradation products (FDPs) and our data showed that the levels of FDPs in IRI rats were significantly higher than that in sham rats, suggesting that fibrinolytic function was activated in IRI models (Figure 7C). And sulodexide pretreatment significantly reduced serum FDPs levels in IRI rats. These results indicated that the beneficial effects of sulodexide on IRI were at least in part on its anti-coagulation properties via activation of ATIII.

### Sulodexide reduced oxidative stress and inhibited apoptosis of HK2 cells under hypoxia/reoxygenation injury

**Figure 8:**
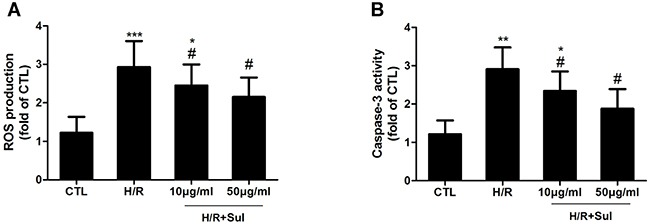
Sulodexide pretreatment protected against hypoxia/ reoxygenation injury in HK-2 cells HK2 cells were cultured to subconfluence, and pretreated with sulodexide (10, 50μg/ml) 30min prior to exposure to hypoxia for 60min, then the cells underwent 30min reoxygenation. **A**. ROS production. **B**. Caspase-3 activity. Data were presented as means ± SD and were representative of three independent experiments.*P<0.05 versus CTL, ^*^P<0.01 versus CTL,^**^*P<0.01 versus CTL; ^#^*P*<0.05 versus H/R.

*In vitro*, the hypoxia/reoxygenation (H/R) injury model for HK2 cells was set up. We found that H/R resulted in a significant increase of intracellular ROS production, which was reduced by sulodexide pretreatment (Figure 8). In addition, sulodexide pretreatment also attenuated H/R induced activation of caspase-3.

## DISCUSSION

In the present study, we sought to assess the therapeutic effects of sulodexide on renal IRI and investigate the potential mechanisms. Our results demonstrated that sulodexide pre-administration attenuated the functional and histologic alterations of kidney in IRI rats, which was accompanied with elevated plasma ATIII activity and blunted oxidative stress, inflammation and apoptosis. Furthermore, sulodexide could directly suppress H/R-induced ROS formation and activation of caspase-3 *in vitro*. Thus, we believe that the reno-protective effects of sulodexide on IRI may be mediated via its anti-oxidation, anti-inflammation, anti-apoptosis and anti-coagulation mechanisms, and sulodexide may represent as a promising therapeutic candidate.

It has been accepted that the disturbance of coagulation systems involved in the pathologic processes of AKI and some antithrombin agents has been proved to be able to accelerate functional recovery of renal function after transient kidney ischemia-reperfusion. In our study, we confirmed that sulodexide, as a potent anticoagulants, also had therapeutic effects on renal IRI in a rat model. In concert with our findings, prior study also had demonstrated that sulodexide could protect against myocardial IRI. It was observed that plasma ATIII activity was elevated in sulodexide-treated IRI rats compared with un-treated IRI rats, indicating the reno-protection by sulodexide might be at least in part dependent on activation of plasma ATIII. This hypothesis can be supported by the following observations: 1) Sulodexide is a potent activator for ATIII [21]; 2) Previous studies proved that exogenous ATIII could protect against renal IRI. Consistently, our previous study also demonstrated that ATIII insufficiency increased the susceptibility to or severity of AKI, suggesting that ATIII is indispensable in the endogenous defense mechanisms against renal IRI; 3) Circulating ATIII activity in sulodexide-pretreated IRI group was increased compared with vehicle-treated IRI at 3h after reperfusion, which preceded the functional and structure changes of kidney between sulodexide-treated and PBS-treated IRI rats. Nonetheless, previous studies have demonstrated that ROS can decrease ATIII activity [22, 23], so we cannot exclude the possibility that sulodexide may inhibit ROS generation thereby inhibiting ischemia-reperfusion induced decrease in ATIII activity. The elevation of ATIII activity conferred by sulodexide could be attributed to the direct activation and subsequent effects of inhibition in ROS production.

In spite of its anti-coagulation property, sulodexide also possesses anti-oxidative effects [15, 16] and anti-inflammatory effects [18, 24, 25]. It has been reported that during the renal IRI, oxidative stress is one of the most critical mechanisms involved in the tubular cellular damage and apoptosis [26, 27]. Our previous studies found that MDA was increased while SOD was increased in rat AKI models [28–30]. Consistently, the present study demonstrated that the improvement of renal function in IRI rats by sulodexide was accompanied with decreased oxidative stress levels. In agreement with our findings, previous studies also demonstrated that the beneficial effect of sulodexide on diabetic nephropathy was associated with the reduction of the MDA levels and enhancement of SOD and catalase activities [31]. Moreover, our *in vitro* studies showed that sulodexide were able to repress intracellular ROS production induced by H/R in HK-2 cells. Thus, on the one hand, the inhibition of oxidative stress by sulodexide *in vivo* could be elucidated by its direct anti-oxidative effects; on the other hand, sulodexide pretreatment increased ATIII activity and subsequently improve the kidney perfusion, which might indirectly inhibit the generation of ROS. Additionally, we observed that sulodexide reduced the expression of pro-inflammatory cytokines in IRI rats. It has been reported sulodexide could inhibit the secretion of inflammatory mediators from lipopolysaccharide-stimulated macrophages *in vitro* [17], and our recent study proved that sulodexide significantly decreased macrophage infiltration in contrast induced nephropathy [32]. In summary, we believed that the anti-inflammatory and anti-oxidative effects of sulodexide contributed to the reno-protection against IRI.

Cumulative evidence has suggested that apoptosis is critically involved in the pathological process in renal IRI [3, 33]. Our study demonstrated that sulodexide treatment significantly alleviated cell apoptosis, which was evidenced by decreased caspase-3 activity and increased Bcl-2 expression. Besides, sulodexide also inhibited the activation of caspase-3 in HK-2 cells under H/R induced injury. In summary, sulodexide might exert its inhibitory effect on apoptosis via a direct inhibition of caspase-3.

The presented data showed that sulodexide protected against renal IRI via activation of ATIII and its anti-oxidative, anti-inflammatory and anti-apoptosis mechanisms. Nonetheless, there was no direct evidence linking the renal protection of sulodexide and activation of ATIII, which was the main limitation of our study. ATIII-knockout rats should be used to investigate the underlying mechanisms of sulodexide in future.

In conclusion, sulodexide can alleviate renal IRI through its anti-oxidative stress and anti-apoptosis, its reno-protective role might be due to its activation for ATIII, indicating that sulodexide may be a potential agent for AKI prevention and treatment. However, whether prophylactic and therapeutic administration of sulodexide can effectively prevent AKI incidence and improve clinical outcome in patient remains to be determined in the future.

## MATERIALS AND METHODS

### Reagents

Sulodexide was purchased from Vessel Due F (Alfa Wassermann, Italy). The primary antibodies, rabbit anti-Bcl-2 and mouse anti-GAPDH were both provided from Cell Signaling Technology (Danvers, MA, USA).

### Animal experimental protocols

This animal experiment in this study was approved by the Animal Care and Ethics Committee of Shanghai Jiao Tong University Affiliated Sixth People's Hospital. Male Sprague-Dawley rats (weighing 250-300g) were purchased from Shanghai Science Academy Animal Center (Shanghai, China).

Animals were randomly divided into 4 groups: sham-operated group treated with tail vein injection of PBS (Sham, n=6); sham-operated group treated with tail vein injection of sulodexide (Sham+Sul, n=6); ischemia-reperfusion group treated with tail vein injection of PBS (IRI, n=6), ischemia-reperfusion group treated with tail vein injection of sulodexide (IRI+Sul, n=6). The model of bilaterally renal ischemia-reperfusion injury was set up as previously described [34]. Sulodexide (2mg/kg, dissolved in 0.1ml PBS), or the same volume of PBS was injected intravenously into the tail vein 30 min before the surgery. Briefly, animals were anesthetized with sodium pentobarbital (50mg/kg). Renal ischemia was induced by clamping both renal pedicles for 45min using a non-traumatic vascular clamp. Animal body temperature was maintained using an animal heating pad. The clamp was removed to restore kidney blood flow. Sham rats underwent the same surgery but without renal pedicle clamping. All the animals were sacrificed 3h or 24h after the surgery, respectively and kidney, blood and urine were collected for further analysis. Blood samples collected from abdominal aorta were moved into one BD Vacutainer® SST™ Serum Separation Tube (Becton-Dickinson, Franklin Lakes, NJ, USA) to obtain serum and one BD Vacutainer® Citrate Tube containing 3.2% buffered sodium citrate (final concentration 0.105mol/L) to obtain plasma, separately. The tubes were centrifuged at 2,000 g for 10min for serum and plasma collection.

### Cell culture and hypoxia/reoxygenation model

Human proximal tubular epithelial cells (HK2 cells, ATCC, Manassas, VA, USA) were cultured in K-SFM at 37°C, 5% CO_2_, supplemented with 5 ng/ml human recombinant EGF and 0.05 mg/ml bovine pituitary extract. Two hours before study, the medium was replaced with glucose-free medium without added growth factors or serum. Cell plates were placed in a glass chamber gassing with 95% N_2_/5% CO_2_ gas and for 60 min followed by reoxygenation (95% 02, 5% CO_2_,) for 30min as previously described [35]. Sulodexide or vehicle (10, 50μg/ml) were added into the medium 30 min before exposed to hypoxia. Cell apoptosis was measured with a Cell Death Detection ELISA kit (Roche Diagnostics, Mannheim, Germany) and intracellular reactive oxygen species (ROS) were detected with a kit (Cell biolabs, San Diego, CA, USA) as previously described [36].

### Assessments of biochemical parameters

Automatic biochemical analyzer (Hitachi7600, Tokyo, Japan) was used to measure blood serum creatinine (Scr) and blood urea nitrogen (BUN) to determine the changes of renal function. ATIII activities in plasma were measured using the commercial kit Accucolor ATIII (SIGMA Diagnostics, Livonia, MI, USA) on an automatic coagulation analysis machine (Sysmex CA7000, SIEMENS, Munich, Germany) as previously described [29]. Fibrinogen degradation products (FDPs) were measured using rat fibrinogen degradation product ELISA Kit (Cusabio, Wuhan, China) following instruction provided by the Manufacturer.

### Histological analyses

The right kidney was fixed and embedded. Paraffin embedded kidney was cut into 3μm sections and subjected to Periodic Acid Schiff (PAS) staining. The histological scoring was evaluated by grading the percentage of affected tubules per 10 randomly chosen, non-overlapping fields (magnification, ×400) in the corticomedullary region according to the following criteria: tubular dilation, loss of brush border, tubular necrosis, and cast formation. The renal injury scoring was estimated on a scale from 0 to 5: 0, none; 1, 0–10%; 2, 11–25%; 3, 26–45%; 4, 46–75% and 5, 76–100%, as described previously [29]. The assessment was performed by an observer who was blind to the study groups. Terminal transferase-mediated dUTP nick-end labeling (TUNEL) staining for cell apoptosis was employed to assess the extent of renal apoptosis in different groups (Roche Diagnostics, Mannheim, Germany), as described previously [37].

### Measurements of oxidative stress markers

The concentrations of malondialdehyde (MDA) and superoxide dismutase (SOD) in renal tissue were measured using commercial kits according to the manufacturer's instruction (Beyotime, Jiangsu, China), and the final levels of MDA and SOD were normalized to the protein concentration of kidney tissue homogenate as previously described [28].

### Measurements of kidney injury markers in blood and urine

Two novel markers of early stage kidney injury, urinary kidney injury molecule-1 (uKIM-1), and serum neutrophil gelatinase-associated lipocalin (sNAGL) were measured using ELISA kits (R&D systems, Minneapolis, MN, USA).

### Quantitative real-time PCR

Total RNA was extracted using Trizol (Invitrogen, Carlsbad, CA, USA) and then, was reverse transcribed into cDNA with GoScript™ Reverse Transcription System (Promega, Madison, WI, USA). Real-time PCR was performed with SYBR^®^ *Premix Ex Taq*^™^ (Tarkara, Dalian, China) by using StepOnePlus PCR Systems (Applied Biosystems, Foster City, CA, USA) as described previously [38]. The specific primers were as following; TNFα: 5′GTCTGTGCCTCAGCCTCTTC3′ (forward) and 5′TGGAACTGATGAGAGGGAGC3′ (reverse); MCP-1: 5′CCCCACTCACCTGCTGCTAC3′ (forward) and 5′CCTGCTGCTGGTGATTCTCTT3′ (reverse); rat ICAM-1 : 5′GAGACCCCGTTGCCTAAA3′ (forward) and 5′CCGCAGGTCCAGTTCAGT-3′ (reverse)’. Quantitation was normalized to the internal control of GAPDH and relative gene expression levels were calculated by the 2^−ΔΔCT^ method.

### Western blot

Protein concentrations of kidney tissue homogenate were measured using BCA assay (Beyotime, Suzhou, Jiangsu, China) and protein samples were separated by 12% sodium dodecyl sulfate-polyacrylamide gels, then were transferred to polyvinylidene difluoride membrane (PVDF) and blocked with 5% non-fat dried milk. The PVDF membranes were then incubated with primary antibody overnight at 4°C and with HRP-conjugated secondary antibodies (Beyotime) for 2h at room temperature. The blotting signals were visualized by the Image Quant LAS 4000 Mini System (GE Healthcare, Pittsburgh, PA, USA). The bands were analyzed using Image J software and GAPDH was used as loading control.

### Statistical analysis

The statistical software SPSS (Ver.18.0) was used for data analysis. One-way ANOVA with Sidak post hoc test or Kruskal-Wallis with Dunn's post test was employed to determine the differences in groups. A value of *P* < 0.05 was considered significant.
